# Postprandial Blood Glucose Outweighs Fasting Blood Glucose and HbA1c in screening Coronary Heart Disease

**DOI:** 10.1038/s41598-017-14152-y

**Published:** 2017-10-27

**Authors:** Jingjing Jiang, Lin Zhao, Liu Lin, Minghui Gui, Qiqige Aleteng, Bingjie Wu, Shanshan Wang, Baishen Pan, Yan Ling, Xin Gao

**Affiliations:** 1 Department of Endocrinology and Metabolism, ZhongShan Hospital, Fudan University, Shanghai, China; 2 Department of Laboratory Medicine, ZhongShan Hospital, Fudan University, Shanghai, China

## Abstract

The objective of the present study is to assess the performance of fasting blood glucose (FBG), postprandial blood glucose (PBG), and glycated hemoglobin (HbA1c) as screening for coronary heart disease (CHD) in an inpatient population undergoing coronary angiography. 1852 consecutive patients scheduled for coronary angiography were classified into Normal Glucose Tolerance (NGT), Impaired Glucose Regulation (IGR), and diabetes, based on FBG, PBG, and HbA1c. Correlations of Gensini score with glucose metabolism and insulin resistance were analyzed. The associations between glycemic variables and Gensini score or the presence of CHD were analyzed by multiple linear regression and logistic regression, respectively. CHD was diagnosed in 488, 622, and 414 patients with NGT, IGR, and diabetes, respectively. Gensini score was positively correlated with FBG (r = 0.09, p < 0.01), PBG (r = 0.20, p < 0.01), and HbA1c (r = 0.19, p < 0.01). Gensini score was not correlated with fasting insulin (r = −0.081, p = 0.36), post-prandial insulin (r = −0.02, p = 0.61), or HOMAIR (r = −0.0059, p = 0.13). When FBG, PBG and HbA1c were pooled altogether, only PBG persisted in its association with Gensini score and the prevalence of CHD. The severity of CHD was associated with glucose rather than insulin resistance in this Chinese population. PBG was optimally correlated with the presence and severity of CHD.

## Introduction

Diabetes has already become a major public health problem in China. According to the recent 2013 national survey, the estimated overall prevalence of diabetes and prediabetes in China was 10.9% and 35.7%, respectively^1^. Current diagnostic cutoff points for diabetes are based on the correlations of glycated hemoglobin (HbA1c), Fasting Blood Glucose (FBG) and Postprandial Blood Glucose (PBG) with the prevalence of diabetic retinopathy, a hallmark of microvascular disease. Nevertheless, macrovascular disease precedes microvascular disease, and cardiovascular disease (CVD) is the leading cause of mortality in patients with diabetes^2^. Conversely, the prevalence of IGT and diabetes in patients scheduled for coronary angiography was much higher than the general population^3^.The association between diabetes and coronary heart disease (CHD) has been well established by numerous studies. Heart disease was listed as the leading cause of death in people with diabetes^4^. The risk for myocardial infarction in diabetic patients without previous myocardial infarction was reported as high as the risk for nondiabetic patients with previous myocardial infarction^5^. About 1 in 6 UKPDS (United Kingdom Prospective Diabetes Study) patients with newly diagnosed type 2 diabetes mellitus had evidence of silent myocardial infarction, increasing the risks of fatal myocardial infarction and all-cause mortality^6^. Significantly increased cardiovascular risk/mortality was also observed even in subjects with IGT^7^. Hence it is important to screen for CHD in patients with glucose dysregulation.

Despite introduction of new radiological modalities like Computed Tomography Angiography (CTA), coronary angiography remains the current gold standard for diagnosing CHD^8^. So far, only a few studies have investigated the correlation between glycemic variables and coronary atherosclerotic status evaluated by coronary angiography. Large-scale studies investigating the correlation between glycemic variables and coronary atherosclerotic status by coronary angiography are still lacking.

The aim of the present study was to evaluate the performance of FPG, PBG, and HbA1c as screening for CHD based on a large inpatient population undergoing coronary angiography.

## Research Design and Methods

### Study population

Our study enrolled a total of 2045 consecutive adults who underwent coronary angiography for suspected CHD in the Cardiology Department of Zhongshan Hospital, Fudan University, a tertiary referral hospital, between March 2013 and November 2013. Patients suspected of CHD in primary and secondary hospitals, based on symptoms like chest pain and dyspnea, were referred to Zhongshan Hospital. In the outpatient department, they were first screened either by routine or dynamic electrocardiogram, coronary computed tomography angiography, exercise treadmill test, or stress myocardial perfusion imaging before coronary angiography assessment. If one of these tests was positive, they were hospitalized and underwent coronary angiography assessment. The exclusion criteria included: acute coronary syndrome, severe systemic diseases, malignancy, and patients with missing data. Finally, 1852 patients were included in the current analysis.

The study was approved by the Ethics Committee of Zhongshan Hospital Fudan University and informed consent was obtained from all participants. All experiments were performed in accordance with relevant guidelines and regulations.

### Data collection

Before coronary angiography, all patients underwent a complete history screening, a physical examination, and biochemical evaluation. Family history of CHD in first-degree relatives, current use of medication, and smoking status were recorded. For female patients, menstrual history was also documented. The BMI was calculated as body weight in kilograms divided by body height in meters squared (kg/m^2^). The WHR (waist-hip ratio) was calculated as the waist circumference at the midpoint between the lower margin of the last palpable rib and the top of the iliac crest, divided by hip circumference at the widest point.

Plasma glucose, lipid profile including serum triglycerides, total cholesterol, HDL cholesterol, LDL cholesterol, ApoA1, ApoB, ApoE and LP(a), glycated albumin, creatinine and uric acid were measured using a biochemical auto analyzer (Hitachi 7600, Japan). Hemoglobin A1c (HbA1c) was determined by HPLC in a National Glycohemoglobin Standardization Program–certified laboratory. Fasting and postprandial serum insulin was measured by an electrochemiluminescence assay (Roche Diagnostics). The index of homeostasis model assessment of insulin resistance (HOMAIR) was calculated as follows: HOMAIR = fasting insulin concentration (mIU/L) × FPG (mmol/L) / 22.5. The index of HOMA β-cell function(HOMAB) was calculated as: HOMAB = 20 × fasting insulin concentration(mIU/L)/[FPG (mmol/L) – 3.5].

### Diagnosis and definition

Patients were classified as Normal Glucose Tolerance (NGT), Impaired Glucose Regulation (IGR), or diabetes based on their glycemic variables. NGT was defined in patients without a previous history of diabetes as having a FPG level < 5.6 mmol/L and a PBG level < 7.8 mmol/l. IGR was defined in patients without a previous history of diabetes as meeting at least one of the following:^1^ FBG ≥ 5.6 mmol/L and < 7.0 mmol/L;^2^ PBG ≥ 7.8 mmol/L and < 11.1 mmol/L;^3^ HbA1c 5.7–6.4%. Diabetes was defined either by a previous history and contemporary hypoglycemic medication or according to the 1999 World Health Organization criteria.

### Coronary angiography

Selective coronary angiography was performed using standard Judkins techniques or a radial approach. Angiographic findings were analyzed by two experienced cardiologists who were blinded to the study protocol. The diagnosis of CHD was defined as having a stenotic lesion of at least 50% in one or more coronary arteries. The severity of stenosis was quantified by the Gensini score^9^.

### Statistical analysis

All statistical analyses were performed by using SPSS for Windows 13.0 (SPSS Inc, Chicago, IL, USA). Continuous variables were presented as mean ± SD and categorical variables were shown in absolute numbers or percentages. Differences between NGT, IGR, and diabetes groups were assessed by Chi-square test for categorical and ANOVA for continuous variables. Correlation between Gensini score and continuous variables was determined by Pearson correlation coefficients. Stepwise adjustments included: 1) age and gender, 2) smoking status, family history of CHD, history of atrial fibrillation, history of hypertension, BMI, WHR, SBP, and DBP, 3) lipid profile and creatinine. Multiple linear regressions were performed to evaluate the associations between Gensini score and glycemic variables. Confounders adjusted in linear regressions included age, gender, smoking status, family history of CHD, history of atrial fibrillation, history of hypertension, BMI, WHR, SBP, DBP, lipid profile, creatinine, and duration of diabetes. Logistic regressions were also performed to evaluate the associations between the presence of coronary heart disease and glycemic variables. In all analysis, *P* < 0.05 was considered statistically significant.

## Result

### General characteristics of the study population

The study population was categorized into three subgroups based on glycemic status. Their characteristics were shown in Table 1. Among the three groups, significant differences were observed in age, BMI, systolic blood pressure, all glycemic variables, insulin levels, lipid profile (triglycerides, HDL-C and APOA1), HOMAIR and finally the presence and severity of CHD.

**Table 1:** Differences in Clinical and Biochemical Characteristics among NGT, IGT and DM patients.

	**TOTAL**	**NGT**	**IGR**	**DM**	**P**
N	1852	623	752	477	
Age (years)	62.76 ± 0.23	61.06 ± 0.41	63.65 ± 0.37	63.57 ± 0.44	< 0.001
Male:Female	1397:455	490:133	557:195	350:127	
Post menopause (n)	331	93	138	100	
Smoke rate	0.4968	0.4992	0.4973	0.4926	NS
CHD rate	0.8229	0.7833	0.8271	86.79	0.002
BMI (kg/m^2^)	24.79 ± 0.07	24.43 ± 0.12	24.82 ± 0.11	25.22 ± 0.14	< 0.001
WHR	0.96 ± 0.01	0.94 ± 0.01	0.97 ± 0.02	0.96 ± 0.01	NS
SBP (mmHg)	128.91 ± 0.33	127.85 ± 0.56	128.12 ± 0.49	131.55 ± 0.68	< 0.001
DBP (mmHg)	77.54 ± 0.21	77.59 ± 0.35	77.58 ± 0.36	77.41 ± 0.37	NS
FBG (mmol/l)	5.71 ± 0.04	4.98 ± 0.02	5.33 ± 0.03	7.29 ± 0.11	< 0.001
PBG (mmol/l)	9.26 ± 0.09	7.04 ± 0.08	8.42 ± 0.10	13.39 ± 0.20	< 0.001
FINS (uIU/ml)	13.46 ± 0.75	9.74 ± 0.48	11.36 ± 0.45	21.78 ± 2.71	< 0.001
PINS (uIU/ml)	81.62 ± 1.85	74.58 ± 2.90	93.03 ± 3.11	72.77 ± 3.51	< 0.001
GA (%)	15.09 ± 0.08	13.23 ± 0.05	14.08 ± 0.07	19.11 ± 0.20	< 0.001
HbA1c (%)	6.19 ± 0.03	5.34 ± 0.01	5.97 ± 0.01	7.66 ± 0.56	< 0.001
TC (mmol/l)	3.92 ± 0.02	3.92 ± 0.04	3.95 ± 0.03	3.89 ± 0.05	NS
TG (mmol/l)	1.78 ± 0.03	1.63 ± 0.04	1.78 ± 0.04	1.99 ± 0.10	< 0.001
LDL (mmol/l)	2.01 ± 0.02	2.02 ± 0.04	2.02 ± 0.03	1.96 ± 0.04	NS
HDL (mmol/l)	1.16 ± 0.01	1.19 ± 0.01	1.16 ± 0.01	1.10 ± 0.01	< 0.001
ApoA1 (mmol/l)	1.21 ± 0.01	1.23 ± 0.01	1.22 ± 0.01	1.19 ± 0.01	0.009
ApoB (mmol/l)	0.70 ± 0.01	0.69 ± 0.01	0.71 ± 0.01	0.71 ± 0.01	NS
ApoE (mmol/l)	35.38 ± 0.40	34.48 ± 0.60	36.01 ± 0.60	35.58 ± 0.93	NS
Lp (a)	393.91 ± 11.19	414.08 ± 19.86	399.30 ± 17.63	359.05 ± 20.97	NS
Scr (umol/l)	80.28 ± 0.52	80.28 ± 0.78	81.46 ± 0.82	78.45 ± 1.16	NS
UA (umol/l)	333.49 ± 2.11	335.59 ± 3.54	341.24 ± 3.28	318.60 ± 4.30	< 0.001
Gensini score	36.72 ± 0.98	30.18 ± 1.39	36.15 ± 1.58	46.15 ± 2.13	< 0.001
HOMAIR	3.83 ± 0.30	2.22 ± 0.13	2.73 ± 0.12	7.75 ± 1.12	< 0.001
HOMAB	213.41 ± 45.90	170.79 ± 20.51	304.64 ± 113.89	130.67 ± 21.67	NS

Abbreviations: CHD, Coronary Heart Disease; SBP, Systolic Blood Pressure; DBP, Diastolic Blood Pressure; BG, blood glucose; FBG, fasting blood glucose; PBG, 2h postprandial blood glucose; FINS, fasting insulin; PINS, 2h postprandial insulin; GA, glycated albumin; HbA1c, Hemoglobin A1C; HDL-C, high-density lipoprotein cholesterol; LDL-C, low-density lipoprotein cholesterol; TC, total cholesterol; TG, triglycerides; Scr, serum creatinine; UA, Uric Acid; BMI, body mass index; WHR, waist hip rate Baseline characteristics of study population was shown in Table 1. Continuous variables were presented as mean ± SD and categorical variables were shown in absolute numbers or percentages. Patients were classified into NGT, IGR and diabetes based on glycemic state according to the 1999 World Health Organization criteria. Chi-square test was used for analysis of categorical variables and ANOVA for continuous variables. NS, not significant.

In the current study population, the prevalence of diabetes and IGR were 25.8% and 40.6%, respectively. Of note, 8.2%(113/1377) of diabetes and 47.9%(659/1377) of IGR were diagnosed for the first time.

### No correlation between insulin resistance and Gensini score

In the present studied population, Gensini score was positively correlated with known risk factors such as age, SBP, non-HDL, ApoB, LP(a), and creatinine; but negatively correlated with HDL and ApoA1(r in Table 2). Gensini score was also positively correlated with PBG, postprandial insulin, glycated albumin, and HbA1c. There was no correlation with HOMA index (HOMAIR or HOMAB).

**Table 2:** The relationship between Gensini score and other Clinical and Biochemical Characteristics.

**Gensini score**	**r**	**P**	**r1**	**P1**	**r2**	**P2**	**r3**	**P3**
FBG	0.05	0.05	0.13	0.00	0.14	0.00	0.09	< 0.001
PBG	0.16	0.00	0.17	0.00	0.17	0.00	0.20	< 0.001
FINS	0.03	0.34	−0.024	0.74	−0.045	0.24	−0.081	0.36
PINS	0.05	0.04	0.01	0.85	−0.015	0.69	−0.02	0.61
GA	0.13	0.00	0.18	0.00	0.18	0.00	0.19	< 0.001
HbA1c	0.13	0.00	0.16	0.00	0.17	0.00	0.19	< 0.001
HOMAIR	0.02	0.58	0.00	0.97	−0.016	0.67	−0.059	0.13
HOMAB	−0.02	0.64	−0.026	0.47	−0.03	0.43	−0.036	0.35

The correlations between Genisi score and other Clinical and Biochemical Characteristics were analyzed using Spearman method. The coefficient of correlation (r) and p value (p) were shown in the table. R1 and P1 represent the coefficient of correlation and p value when adjusted for correlations were adjusted for age & gentle; R2 and P2 when adjusted for Age & gentle & smoke history & CHD history & atrial fibrillation & hypertension & BMI & WHR & SBP & DBP: R3 and P3 when adusted for Age & gentle & smoke history & CHD history & atrial fibrillation & hypertension & BMI & WHR & SBP & DBP & TC & Tg & LDL & HDL & non-HDL & Apo A1 & Apo B & Apo E & Lp(a) & Scr.

After adjusting for age and gender, FBG also became positively correlated with Gensini score, while postprandial insulin lost its correlation with Gensini score (r1 in Table 2). The positive correlation with four glycemic variables persisted after adjusting smoking status, family history of CHD, history of atrial fibrillation, history of hypertension, BMI, WHR, SBP, DBP (r2 in Table 2), and further adjustments of lipid profile and creatinine (r3 in Table 2). Gensini score was not correlated with insulin (fasting or post-prandial), HOMAIR (representing insulin resistance) or HOMAB (representing β cell function).

### Association of FPG, PPG and HbA1c with CHD

In multiple linear regression analysis, Gensini score was associated with FBG (β = 0.062, p = 0.018), PBG (β = 0.163, p < 0.001), and HbA1c (β = 0.107, p < 0.001). Nevertheless, when pooled altogether, only PBG remained significantly and independently associated with the Gensini score (Table 3). In logistic regression analysis, the presence of CHD was associated with FBG (B = 1.155, p = 0.008), PBG (β = 1.098, p < 0.001), and HbA1c (β = 1.239, p = 0.002). When pooled altogether, the association only remained significant for PBG (β = 1.096, p = 0.000) (Table 4).

**Table 3:** Multiple linear regression.

**Gensini**		**Standardized Coefficients**	**95.0% Confidence Interval for B**		**P value**
			Lower Bound	Upper Bound	
Model 1 (FBG)	HDL	−0.077	−16.546	−3.420	0.003
	Duration of DM	0.090	0.332	1.181	< 0.001
	creatinine	0.078	0.052	0.243	0.002
	Lp(a)	0.073	0.002	0.010	0.002
	FBG	0.062	0.281	2.965	0.018
	Gender	0.065	1.193	11.312	0.015
	ApoB	0.057	1.982	20.214	0.017
Model 2 (PBG)	PBG	0.163	1.219	2.300	< 0.001
	apoA1	−0.099	−25.965	−7.686	< 0.001
	creatinine	0.068	0.027	0.224	0.013
	Lp(a)	0.077	0.002	0.011	0.003
	ApoB	0.064	2.604	21.743	0.013
	Gender	0.059	0.270	10.968	0.040
Model 3 (HbA1c)	HbA1c	0.107	1.906	6.031	< 0.001
	apoA1	−0.076	−21.333	−4.195	0.004
	creatinine	0.073	0.044	0.234	0.004
	Lp(a)	0.071	0.002	0.010	0.003
	ApoB	0.063	2.936	21.260	0.010
	Gender	0.063	1.001	11.168	0.019
	Duration of DM	0.056	0.005	0.935	0.048
Model 4 (FBG, PBG, HbA1c)	PBG	0.163	1.219	2.302	< 0.001
	ApoA1	−0.099	−25.987	−7.631	< 0.001
	creatinine	0.068	0.027	0.224	0.013
	Lp(a)	0.076	0.002	0.011	0.003
	ApoB	0.064	2.712	21.899	0.012
	Gender	0.059	0.274	10.984	0.039

Multiple linear regression was performed to identify the parameters associated with the CHD. In model 1, only fasting plasma glucose (FPG) was taken into account as glycemic parameter with other variables. In model 2, only postprandial blood glucose (PBG) was taken into account as glycemic parameter with other variables.In model 3, only HbA1c was taken into account as glycemic parameter with other variables. In model 4, FPG, PBG and HbA1c were all taken into account as glycemic parameters with other variables.

**Table 4:** Multiple Logistic regression between CHD with FBG, PBG and HbA1c.

**CHD**		**P value**.	**Exp(B)**	**95% C.I.for EXP(B)**	
				Lower	Upper
Model 1 (FBG)	Age	<0.001	1.029	1.015	1.043
	Gender	<0.001	2.813	2.083	3.799
	SBP	0.032	1.011	1.001	1.021
	histrory of Af	0.028	0.515	0.284	0.932
	HDL	0.035	0.634	0.415	0.968
	Lp(a)	<0.001	1.001	1.000	1.001
	FBG	0.008	1.155	1.039	1.284
Model 2 (PBG)	age	0.001	1.025	1.010	1.039
	Gender	<0.001	3.179	2.357	4.287
	Lp(a)	0.003	1.001	1.000	1.001
	PBG	<0.001	1.098	1.051	1.147
Model 3 (HbA1c)	Age	<0.001	1.028	1.014	1.042
	Gender	<0.001	2.809	2.079	3.795
	SBP	0.033	1.011	1.001	1.021
	histrory of Af	0.025	0.507	0.280	0.918
	HDL	0.045	0.647	0.423	0.990
	Lp(a)	<0.001	1.001	1.000	1.001
	HbA1c	0.002	1.239	1.078	1.424
Model 4 (FBG, PBG, HbA1c)	Age	0.001	1.024	1.010	1.039
	Gender	<0.001	3.139	2.326	4.236
	Lp(a)	0.002	1.001	1.000	1.001
	PBG	<0.001	1.096	1.050	1.145

Multiple logistic regression was performed to identify risk factors associated with the CHD. In model 1, only fasting plasma glucose (FPG) was taken into account as glycemic parameter with other variables. In model 2, only postprandial blood glucose (PBG) was taken into account as glycemic parameter with other variables.In model 3, only HbA1c was taken into account as glycemic parameter with other variables. In model 4, FPG, PBG and HbA1c were all taken into account as glycemic parameters with other variables. Exp, exponential; B, coefficient; CI, confidence interval.

## Discussion

The prevalence of diabetes was 25.8% in this study, 2.37 fold higher than the 2013 nationwide survey, which estimated the prevalence of diabetes as 10.9%^1^. In the studied population, the severity of coronary stenosis, quantified as Gensini score, was positively correlated with FBG, PBG, glycated albumin, and HbA1c even after adjustments for all known factors. In contrast, no correlation of Gensini score with fasting insulin, postprandial insulin, HOMAIR or HOMAB remained significant after adjustments for age and gender. Two previous studies in Caucasian men, including 363 men without a diabetes history and 234 men with NGT, observed significant correlations between the number of involved vessels and postload glycemia, HbA1c, fasting insulin, and postload insulin, suggesting that the severity of atherosclerosis was positively correlated with both glucose and insulin resistance^10,11^. Nevertheless, this was not supported by the findings of the present study. Although hyperinsulinemia or insulin resistance has been regarded as a risk factor for developing cardiovascular disease^12–14^, most East Asian patients with diabetes have much more moderate BMI compared with Caucasians. Impaired insulin secretion was reported to contribute more to the incidence of diabetes than insulin resistance^15^. Therefore, the significance of insulin resistance in the development of CHD might also be different from that of Caucasians. In fact, our result was in agreement with a previous Korean study of 230 patients, which also concluded that postchallenge hyperglycemia, but not hyperinsulinemia, was associated with CHD assessed by angiography^16^. The UKPDS study reported that in patients with type 2 diabetes, each 1% reduction in mean HbA1c was associated with a 14% reduction in the risk of myocardial infarction^17^.

Hyperglycemia itself is an independent risk factor for cardiovascular diseases. Therapies targeting postprandial glucose, including acarbose and insulin, prevent CVD in diabetic patients^18,19^, whose beneficial effects are independent of improving insulin resistance. It is generally accepted that hyperglycemia leads to atherosclerosis via mechanisms unified as overproduction of ROS and consequent oxidative stress^20^. Recently more mechanisms have been proposed. Firstly, hyperglycemia can cause epigenetic changes, especially dysregulation of microRNAs. These microRNA changes can lead to dysfunction of endothelial cells, vascular smooth muscle cells, platelets, and macrophage, as well as abnormal lipid metabolism, all of which are involved in atherosclerosis^21–23^. Secondly, the ROS overproduction caused by hyperglycemia triggers redox modifications and malfunction of ion channels in cardiomyocytes, e.g. the type 2 ryanodine receptor (RyR2) on the endoplasmic reticulum (ER). This direct effect may worsen the cardiovascular function observed in chronic hyperglycemia. Thirdly, hyperglycemia alters signaling pathways in atherosclerotic plaques. Plaques from patients with diabetes had more NF-kB expression and less SIRT6 expression, indicating a less stable plaque phenotype^24–27^. Collectively, these studies highlighted the contributions of hyperglycemia to the development of cardiovascular injury in diabetes.

The outstanding importance of PBG was supported by both linear and logistic regression analyses. PBG had the most significant correlation with prevalence and severity of CHD amongst the three glycemic parameters. The effects of FBG and HbA1c were completely masked by PBG when they were simultaneously included in the analysis. In logistic regression analysis, PBG stands in line with well-known risk factors like age, gender, and LP(a). Although HbA1c depicted a chronic glycemic profile, glucose fluctuations during postprandial periods triggered oxidative stress more than chronic sustained hyperglycemia. On the contrary, fasting hyperglycemia played a major role as soon as the HbA1c level rises above 8.4%^28^. Postprandial glucose was more sensitive than HbA1c in screening for prediabetes. 2-h Glucose level and IGT were stronger predictors of CVD than HbA1c^29^. Kataoka *et al*.^30^ reported that diffuse coronary artery narrowing (calculated by averaged vessel diameter and lesion length) was associated with postprandial hyperglycemia in 534 Japanese patients using quantitative coronary angiography. Later in a larger Caucasian cohort of 1040 patients, Saely *et al*.^31^ reported that PBG was associated with the number of significant coronary stenoses and the Gensini score. These results were consistent with our findings that PBG was associated with Gensini score, reiterating the importance of PBG in the natural development of coronary artery disease.

Another important finding in the present study was the correlation of PBG with the severity of atherosclerosis (quantified by Gensini score) was independent of the duration of diabetes. This finding may have important clinical implications. The duration of diabetes is known to contribute significantly to CVD risks. A 1.38-fold increased risk for CHD and a 1.86-fold higher risk for CVD death has been reported for each 10-year increase in duration of diabetes by the Framingham Heart Study^32^. Although the macrovascular complications of diabetes increase with duration, based on our findings, we suggest PBG should be closely monitored for early identification of CHD patients. In other words, aggressive screening of CHD is justified, provided that PBG is elevated, whether diabetes is newly diagnosed or diagnosed long ago.

The strengths of our study resided in the large study population and the use of the “gold standard” coronary angiography for assessing coronary stenosis. However, this study also had some important limitations: 1) As a cross-sectional study, we were unable to establish any causal relationships. 2) Our study focused on a highly selected group of patients, i.e. symptomatic inpatients. We didn’t have an independent population which was more general and less severe to validate the findings. 3) Also, the study involved only Asians, therefore the results may not be generalized to other racial or ethnic groups. 4)The female sample size was relatively small and thus did not allow separate analyses by gender. 5) The superiority of PBG was not validated in another independent population.

In conclusion, the severity of CHD was associated with glucose rather than insulin resistance in a large Chinese inpatient population scheduled for coronary angiography. Postprandial hyperglycemia was independently correlated with the presence and severity of coronary atherosclerosis in this population. These results suggest that the timing of screening should be based on postprandial glucose level, which outperformed FBG, HbA1c and insulin levels. Nevertheless, these findings still need to be validated in another independent population if we want to extend the significance of current study to a more general scenario. Follow-up studies are also needed to investigate the predictive power of postprandial glucose for future cardiovascular events.
